# Barriers to and facilitators of implementing obstructive sleep apnea screening in stroke patients: a scoping review protocol

**DOI:** 10.3389/fneur.2025.1690372

**Published:** 2025-10-16

**Authors:** Yashi Lin, Jiali Zhao, Wenhui Xiao, Fang Ding, Zhen Fang, Liang Guo, Shuhui Lou

**Affiliations:** ^1^School of Nursing, Zhejiang Chinese Medical University, Hangzhou, Zhejiang, China; ^2^Department of Neurology, The Second Affiliated Hospital of Zhejiang Chinese Medical University (The Xin Hua Hospital of Zhejiang Province), Hangzhou, Zhejiang, China; ^3^Department of Rehabilitation, The Third Affiliated Hospital of Zhejiang Chinese Medical University, Hangzhou, Zhejiang, China; ^4^Department of Neurosurgery, Tongde Hospital of Zhejiang Province, Hangzhou, Zhejiang, China

**Keywords:** obstructive sleep apnea, stroke, sleep disorders screening, barriers and facilitators, scoping review protocol, ottawa model of research use

## Abstract

**Introduction:**

Obstructive sleep apnea (OSA) is a highly prevalent but frequently undiagnosed sleep disorder among stroke patients. It is associated with increased risks of stroke recurrence, reduced rehabilitation effectiveness, and elevated mortality. Despite guideline recommendations for routine OSA screening in stroke care, implementation remains inconsistent in clinical practice. As a modifiable sleep-related risk factor with significant implications for neurological outcomes, better integration of OSA screening in post-stroke care is urgently needed. Thus, this scoping review protocol outlines a systematic approach to identifying barriers to and facilitators of OSA screening in stroke populations.

**Methods and analysis:**

This scoping review will follow the methodological framework provided by the Joanna Briggs Institute (JBI) and will be reported using Preferred Reporting Items for Systematic Reviews and Meta-Analyses extension for Scoping Reviews (PRISMA-ScR) guidelines. The search will be performed on CNKI, WanFang, SinoMed, PubMed, Embase, Web of Science, the Cochrane Library, CINAHL, ProQuest Dissertations, OpenGrey, and Google Scholar. Targeted searches of international organization websites will also be conducted. No restrictions will be imposed based on study design or year of publication. Data will be synthesized using the content analysis approach and mapped onto the Ottawa Model of Research Use (OMRU), including domains such as evidence-based innovation, potential adopters, practice environment, implementation process, and adoption outcomes.

**Discussion:**

The findings are expected to inform future research and support the integration of sleep disorder screening into stroke care pathways. Ultimately, the review will improve stroke outcomes by addressing sleep health as a critical but often overlooked component of post-stroke management.

**Scoping review registration:**

Open science framework (osf.io/tb7z8).

## 1 Introduction

Stroke is defined as a neurological deficit resulting from acute focal injury due to an interruption or reduction in cerebral blood flow (1). It is broadly classified into ischemic and hemorrhagic types (2), whereas its acute clinical manifestation exhibits significant heterogeneity. This complexity necessitates further etiological stratification, such as cardioembolic stroke, lacunar infarction, and atherothrombotic infarction for ischemic strokes, alongside intracerebral hemorrhage for hemorrhagic strokes (3). Accurate differentiation of stroke subtypes is clinically important, as they are associated with distinct risk profiles, initial severities, and prognostic trajectories, all of which influence management strategies (3). According to the 2019 Global Burden of Disease (GBD) Study, stroke remains the second leading cause of global deaths (4). Annually, approximately 12.2 million new stroke cases are reported. Moreover, 101 million individuals live with stroke-related conditions, and 6.55 million deaths are attributed to stroke (4).

Sleep-related breathing disorders (SRBDs), particularly obstructive sleep apnea (OSA), have been increasingly recognized as significant contributors to both the onset of first-ever ischemic stroke and the risk of recurrence. SRBDs are also associated with poorer clinical outcomes, including increased stroke severity, prolonged hospitalization, and reduced functional recovery (5). Among SRBDs, OSA is the most prevalent condition in stroke patients (5). Post-stroke neuromuscular dyscoordination, particularly involving the upper airway, intercostal, and diaphragmatic muscles, can predispose individuals to OSA (6). Notably, OSA is a disorder characterized by recurrent pharyngeal airway collapse during sleep, resulting in repeated arousals, fragmented sleep patterns, and pronounced daytime sleepiness (7). A meta-analysis reported that 72% of stroke patients exhibit an apnea-hypopnea index (AHI) greater than 5 (8) and a prevalence significantly higher than the 35% observed in the general population (9). Additionally, the prevalence of post-stroke OSA has recently shown a rising trend (10). This is particularly concerning given that comorbid OSA is associated with an increased rate of stroke recurrence and all-cause mortality (11). Consequently, a 10-year cohort study reported a 75% higher mortality risk in stroke patients with concomitant OSA compared to those without sleep apnea (12). In recognition of these risks, the American Heart Association and the American Stroke Association issued a 2021 guideline recommending that patients with ischemic stroke or transient ischemic attack (TIA) be considered for OSA evaluation (Class 2b, Level of Evidence B-NR) (13).

The prevalence of OSA varies across different stages of stroke recovery (14). Research shows that OSA is more common during the acute and subacute phases of stroke compared to the chronic phase, with reported prevalence rates of 68.4% in the acute phase, 71.3% in the subacute phase, and 60.6% in the chronic phase (15). These fluctuations likely reflect the time-dependent nature of post-stroke impairments in respiratory regulation, including abnormal breathing patterns and reduced respiratory muscle tone, which often stabilize after the subacute period (16).

However, some studies have found no statistically significant differences in OSA prevalence between the acute and chronic phases (8). This discrepancy may arise from variations in diagnostic criteria, timing of assessment, or the natural resolution of symptoms in patients with milder cases (17). Despite these inconsistencies, OSA can persist and continue to negatively impact recovery throughout all phases of stroke rehabilitation– from acute to chronic. Therefore, early detection and timely intervention for OSA may be crucial in improving post-stroke recovery and reducing associated complications (18, 19).

Screening for OSA is critical in stroke care, as it can help prevent serious complications and optimize treatment strategies, as OSA is recognized as a predictor of poor post-stroke outcomes (20). Observational studies have indicated that untreated OSA following a stroke is linked to an increased risk of stroke recurrence (21), higher mortality rates (12, 22, 23), reduced effectiveness of rehabilitation, and prolonged hospitalization (24). Thus, early identification and management of post-stroke OSA may improve clinical outcomes. For example, screening for OSA and initiating treatment during the acute phase of a stroke may enhance recovery by preserving the ischemic penumbra and improving cerebral perfusion (25). Continuous positive airway pressure (CPAP) therapy is the first-line treatment for patients with moderate to severe OSA after a stroke (26). Moreover, evidence suggests that initiating CPAP therapy within the first 48 h of stroke onset leads to better neurological outcomes compared to delayed initiation (27, 28). Additionally, CPAP therapy has been shown to improve short-term outcomes, such as significant reductions in the AHI (22), improved sleep quality, decreased daytime sleepiness (21), reduced snoring, and enhanced cognitive function (29). Thus, the evidence strongly supports the incorporation of OSA screening into routine post-stroke management, with treatments tailored according to the severity of the condition and any comorbidities present (20, 30).

Despite its clinical significance, screening for OSA in post-stroke patients remains infrequent. One study found that 22% of stroke patients were diagnosed with OSA within 15 days of onset, yet many had not been screened for the condition (31). Additionally, research by Brown et al. (32) revealed that only 6% of stroke patients in the United States were tested for sleep apnea after experiencing a stroke. To address this issue, Johnson et al. (33) examined the ongoing debate surrounding the necessity of screening for this prevalent but treatable condition. They attributed the reluctance to trade-offs involving assessment time, the selection and validity of diagnostic tools, and uncertainties regarding the effectiveness of treatment. Nevertheless, given the growing evidence, OSA should be systematically screened for and managed as a routine component of post-stroke care.

A previous review by Swartz et al. (34) examined barriers to implementing screening and management strategies for depression, OSA, and cognitive impairment screening in post-stroke patients. The study proposed an innovative framework that conceptualizes the interconnectedness of post-stroke depression, OSA, and cognitive dysfunction as “DOC.” It systematically identified various multidimensional barriers to the implementation of these strategies in clinical practice. These included factors related to the screening tools, the practice environment, and the characteristics of potential adopters. However, the review primarily focused on the broader rationale and challenges associated with screening for DOC comorbidities as a group rather than delving into issues specific to OSA. Additionally, it did not address key challenges unique to OSA screening in stroke care, such as the absence of standardized screening guidelines. Furthermore, our comprehensive search of multiple databases did not identify any existing systematic or scoping reviews that specifically evaluate the barriers to and facilitators of OSA screening in post-stroke populations.

Existing evidence indicates that the evaluation of OSA signs and symptoms should be conducted in all stroke patients (33). However, OSA is frequently underrecognized as a modifiable risk factor for stroke recurrence (35), leading to inadequate post-stroke recovery outcomes. A comprehensive understanding of the facilitators and barriers to OSA screening in this population may aid the development of large-scale screening strategies as part of routine stroke care. Notably, such insights could also guide the design of more effective screening and diagnostic tools, ultimately improving the management of stroke-related OSA. Therefore, this scoping review, guided by the Joanna Briggs Institute’s methodological framework (36), aims to identify and synthesize the barriers to and facilitators of OSA screening in stroke patients. Ultimately, the goal is to promote the integration of evidence-based assessment practices into clinical care.

## 2 Review question

What are the barriers and facilitators to implementing screening for OSA in stroke patients?

## 3 Methods and analysis

### 3.1 Inclusion and exclusion criteria

To ensure scientific rigor, reproducibility, and applicability, this scoping review will follow the methodological framework established by the Joanna Briggs Institute (JBI) (36). The review will also adhere to the Preferred Reporting Items for Systematic Reviews and Meta-Analyses extension for Scoping Reviews (PRISMA-ScR) checklist (37) (see Supplementary Additional File 1) to promote transparency and standardized reporting. Given the limited research available, the review will include a wide range of evidence sources, including original research studies (quantitative, qualitative, and mixed-methods), reviews, clinical practice guidelines, expert opinions, and conference abstracts or proceedings. No restrictions will be imposed based on study design or year of publication. The review will be guided by the Population, Concept, Context framework described below and aligned with the study’s objectives and research questions.

#### 3.1.1 Participants

This review will include studies involving populations relevant to the influence and implementation of OSA screening in stroke care. Eligible participants may include (i) stroke patients at any stage of recovery (acute, subacute, or chronic), regardless of age, gender, stroke type (ischemic or hemorrhagic), or sociodemographic characteristics; (ii) family members involved in decision-making regarding OSA screening for stroke patients; (iii) healthcare professionals directly engaged in the screening, assessment, or implementation of OSA screening in stroke care, such as neurologists, stroke nurses, sleep specialists, and primary care providers; and (iv) policymakers or healthcare administrators involved in the development, implementation, or evaluation of OSA screening policies or strategies within stroke care pathways.

Exclusion criteria include studies involving (i) individuals with sleep disorders other than OSA (e.g., narcolepsy); (ii) patients receiving only palliative, hospice, or comfort care; and (iii) healthcare professionals or policymakers not directly involved in stroke-related OSA screening or management.

#### 3.1.2 Concept

This review aims to identify and synthesize barriers and facilitators of OSA screening in stroke patients and related populations involved in the screening process. The concept encompasses a wide range of factors that influence the implementation of screening, including but not limited to:

(i) The use and accessibility of screening tools (e.g., questionnaires, polysomnography);
(ii) Characteristics of the screening process (e.g., timing, integration into care pathways);
(iii) Healthcare provider’s knowledge, skills, and attitudes toward OSA screening;
(iv) Policy or institutional support for screening programs;
(v) Patient acceptance, engagement, and adherence to screening and follow-up;
(vi) Indirect influences such as perceived or actual treatment outcomes affecting screening uptake.

Studies that focus solely on OSA treatment modalities or the underlying pathophysiological mechanisms, without addressing issues related to screening implementation, will be excluded.

#### 3.1.3 Context

The context of this review will include a range of healthcare settings where stroke patients may undergo screening for OSA, including hospitals, rehabilitation centers, outpatient clinics, and community health settings. Studies will be included if they report on OSA screening practices conducted during hospitalization, rehabilitation, or community-based stroke management. Studies without accessible full texts will be excluded.

#### 3.1.4 Evidence sources

All types of studies will be included, including original research, reviews, guidelines, and conference proceedings.

### 3.2 Search strategy

To identify relevant published literature, a comprehensive search will be conducted across multiple electronic databases and platforms. Complete search strategies for PubMed, CINAHL, and CNKI are provided in Supplementary Additional File 2 and will serve as the basis for adapting searches for other databases, including WanFang, SinoMed, Embase (via Ovid), Web of Science, and the Cochrane Library. The search strategy was collaboratively developed and critically reviewed in consultation with a health sciences librarian with expertise in academic literature retrieval. The search will incorporate controlled vocabulary (e.g., MeSH terms) and free-text keywords related to the core concepts of stroke, obstructive sleep apnea, screening, and influencing factors. Boolean operators will be used to structure search queries tailored to each database’s indexing terms and syntax. No restrictions will be placed on the publication date or language.

In addition to database searches, gray literature sources will be consulted to identify unpublished or non-indexed studies. These sources will include ProQuest Dissertations, OpenGrey, and Google Scholar. Details of the Google Scholar search strategy, including search terms, number of records retrieved (limited to the first 200 results per query), and exclusion criteria, are provided in the Supplementary Additional File 2. Targeted searches of international organization websites will also be conducted to identify relevant policy documents, clinical guidelines, and reports. These will include materials from the World Stroke Organization, the American Heart Association/American Stroke Association, the American Academy of Sleep Medicine, the European Sleep Research Society, and the World Health Organization. Conference abstracts and proceedings will be included, drawing from both electronic databases and platforms in addition to Google Scholar. No date or language restrictions will be applied.

To minimize language bias, records published in languages other than English or Chinese will still be captured during the search process. Titles and abstracts will be screened using an automated translation tool (Google Translate). Full-text articles that meet the inclusion criteria will be translated with the assistance of professional translation services or qualified language experts.

Additionally, the reference lists of all included studies will be manually screened to capture any additional relevant literature that may not have been identified through the electronic searches. This multi-pronged approach is designed to ensure a comprehensive and systematic collection of all pertinent evidence.

### 3.3 Source of evidence selection

NoteExpress will be used to manage the retrieved literature and remove duplicates. Rayyan software (38) will be used to facilitate collaborative screening and streamline the review process. Two trained reviewers will independently conduct an initial screening of titles and abstracts, followed by the full-text screening of potentially relevant studies based on predefined inclusion and exclusion criteria. Discrepancies will be resolved through discussion, with the involvement of a third reviewer, if a consensus cannot be reached.

For each conference abstract or non-peer-reviewed record, efforts will be made to identify a corresponding full-text publication. All available information will be extracted, and the completeness of the data will be documented. If essential data are missing, the corresponding or presenting author will be contacted for clarification. If no response is received within 7 days, a follow-up email will be sent. Records with unresolved missing data after an additional 7 days will be excluded from the review.

The study selection process will be documented and reported following the PRISMA-ScR checklist and explanation (37) (see Supplementary Additional File 3). Prior to the formal screening, a pilot test will be conducted on a random sample of 200 records. During this phase, the review team will discuss any disagreements and refine the inclusion criteria and operational definitions as necessary. Formal screening will begin once ≥80% agreement between reviewers is achieved.

### 3.4 Data extraction

A standardized data extraction form will be used to systematically organize the included literature and ensure completeness and comparability of the data. The extracted information consists of basic study characteristics (Table 1), such as authorship, year, country, publication type, study design, population, aims, settings (stroke unit, rehabilitation, outpatient, etc.), OSA ascertainment method (screening questionnaire, portable monitoring, or polysomnography), timing of screening relative to stroke onset (acute/subacute/chronic; days/weeks), personnel performing the screening (e.g., nurse, physician, sleep specialist, or research staff), key barriers/facilitators, and notes on missing data or contact attempts with study authors. Factors related to barriers and facilitators will be extracted descriptively during the data extraction phase. These factors will later be categorized and mapped to the OMRU domains during the analysis phase (see Table 2).

**Table 1:** Characteristics of included studies.

Study information (authors, year, country, publication type)	Study design	Study population	Aims	Settings	OSA ascertainment method	Timing relative to stroke onset	Personnel performing the screening	Key barriers/facilitators	Notes on missing data/contact attempts
1									
2									
3									

**Table 2:** Barriers and facilitators — extraction and mapping using the OMRU framework.

OMRU element	Factor description (e.g., tools, training, policies, knowledge)	Classification (facilitator/barrier)	Extracted examples (specific facilitators or barriers described in the study)	Source(s)
Evidence-Based innovation				
Practice environment				
Potential adopters				
Implementation process				
Outcome of adoption				

To ensure reliability, the data extraction form will be piloted on 10% of the included studies. If frequent discrepancies are identified, the extraction instructions and definitions will be refined before full data extraction begins. Data will be extracted independently by two reviewers. Any disagreements will be resolved through discussion; if consensus cannot be reached, a third reviewer will be consulted for arbitration.

A methodological quality appraisal of the included studies will not be conducted, in accordance with established scoping review methodology. The primary aim of this review is to map the existing evidence and identify barriers and facilitators of OSA screening, rather than to assess the risk of bias in individual studies (36). This approach aligns with the latest JBI guidance and other published methodological recommendations for conducting scoping (36, 39).

### 3.5 Data analysis and presentation

Following data extraction, the analysis and synthesis will be guided by the OMRU (40), a widely applied framework for identifying and categorizing barriers and facilitators in healthcare implementation research (41). Importantly, OMRU provides a structured, multi-level approach to examining factors that influence the implementation of post-stroke OSA screening. As such, this encompasses dimensions such as research evidence, potential adopters, practice environment, implementation processes, and adoption outcomes.

A directed content analysis will be employed to map the extracted data onto the key domains of the OMRU framework (42). This approach is well-suited to implementation research, as it enables the systematic classification of barriers and facilitators using established theoretical constructs, while also allowing for the identification of novel factors that may emerge from the data.

Evidence-based innovation: Evaluation of various OSA screening tools’ validity, reliability, and clinical applicability.

Potential adopters: Assessment of healthcare providers’ awareness, attitudes, and competencies in OSA screening, as well as patient acceptance and adherence to screening recommendations.

Practice environment: Analysis of institutional and organizational factors, including resource availability, hospital policies, and interdisciplinary collaboration, that influence the feasibility of screening.

Implementation process: Identification of barriers and facilitators related to workflow integration, timing of screening, and coordination among care teams within stroke services.

Outcomes of adoption: Examination of screening uptake, influence on clinical decision-making, and potential long-term impacts on patient outcomes.

For qualitative data, two trained reviewers (coders) will independently code the extracted information using NVivo software. An initial coding framework will be developed deductively based on OMRU domains. During the coding process, open coding will also be applied inductively to capture any emerging themes that are not encompassed within the predefined OMRU domains.

Before full coding begins, a pilot coding phase will be conducted on approximately 15–20% of the data to refine the coding guidelines and ensure a shared understanding of coding rules. During this phase, inter-coder agreement will be assessed, with a target of at least 80% agreement between coders. To address discrepancies, coders will meet regularly to review differences, and unresolved issues will be resolved through discussion and consensus, with a third senior coder available for adjudication if needed. This process will be maintained throughout both the pilot and main coding phases to ensure consistent application of final codes. All key decisions and modifications to the coding framework will be documented to ensure transparency and methodological rigor.

Quantitative indicators, such as reported screening rates, adherence statistics, and institutional policy metrics, will be summarized descriptively using measures such as the median, interquartile range, and range. Where conceptually aligned, these quantitative findings will be mapped to OMRU domains and integrated with qualitative data to provide a comprehensive overview. As a final step, the synthesis will provide a structured summary of barriers and facilitators by OMRU domain, supported by exemplar quotes or data points, and will highlight priority areas for future research.

### 3.6 Stakeholder consultation

In accordance with JBI methodology (36), stakeholder consultation is considered an optional stage in scoping reviews. While such engagement can enrich the interpretation of findings and enhance their relevance, this review will not include a formal stakeholder consultation due to practical constraints. These include limited time and resources, as well as the geographic dispersion of key stakeholders across institutions. Conducting a meaningful consultation would require careful planning, recruitment of diverse participants (e.g., clinicians, patients, policymakers), and prior ethical approval, all of which fall outside the scope of the current study. Future research will prioritize stakeholder involvement through interviews or focus groups to complement the literature-based findings and address real-world implementation challenges not captured in published sources.

## 4 Patient and public involvement

No patients or members of the public will be involved in the design, conduct, reporting, or dissemination of this scoping review.

## 5 Ethics and dissemination

Ethical approval is not required for this scoping review, as it analyzes data from publicly available sources and does not involve human participants. The findings will be disseminated through multiple targeted strategies. In addition to publication in peer-reviewed journals and presentations at academic conferences, results will be shared with stroke care networks, sleep medicine societies, and professional organizations involved in neurological rehabilitation to promote awareness and support the implementation of OSA screening. Policy briefs and practical checklists summarizing key barriers and facilitators will be developed for stroke units and hospital administrators to facilitate the integration of evidence into clinical practice. Potential end-users of this work include clinicians, nurse specialists, rehabilitation teams, and health policy makers involved in post-stroke care.

## 6 Discussion

Establishing a detailed and pre-defined protocol for this scoping review enhances methodological transparency and rigor, which helps minimize the risk of reporting bias. To the best of our knowledge, this will be the first scoping review to systematically map the literature on barriers to and facilitators of OSA screening in stroke populations, utilizing the OMRU framework as a guiding reference. By applying a multidimensional conceptual model, the review aims to capture the complex factors that influence the screening practices in real-world clinical settings.

Given the increasing recognition of sleep disorders as critical yet underdiagnosed contributors to neurological outcomes, this review will provide timely insight into how sleep-related conditions, particularly OSA, are addressed in the stroke care continuum. Thus, a better understanding of the challenges and factors that facilitate implementation may clarify the role of sleep health in post-stroke recovery and long-term prognosis. The findings are expected to identify gaps between evidence-based screening recommendations and current clinical practices, informing future research and practical strategies to improve screening uptake.

Beyond the scope of this review, future research should investigate the comparative effectiveness of various OSA screening tools in stroke populations, as well as strategies for integrating screening into multidisciplinary stroke care pathways. Such efforts are essential to generate evidence for developing standardized and scalable screening protocols. Additionally, future studies should examine how clinical heterogeneity among stroke subtypes may influence the relationship between stroke and OSA. Notably, given the distinct pathophysiological mechanisms, prognostic implications, and clinical characteristics of lacunar versus non-lacunar ischemic strokes, it is critical to assess whether these differences affect the prevalence, clinical presentation, and management of post-stroke OSA (43). Ultimately, this review may contribute to more integrated and responsive models of post-stroke care, where the identification and management of sleep-disordered breathing are recognized as essential components of secondary prevention and neurorehabilitation.

## 7 Strengths and limitations

This scoping review will follow the methodological framework established by the JBI and adhere to the PRISMA-ScR guidelines, thereby ensuring methodological rigor, transparency, and reproducibility. A key strength of this review is the application of a well-established implementation science framework – the OMRU – which will guide the categorization of barriers and facilitators, thereby enhancing the practical relevance of the findings in both clinical and policy contexts. Additionally, a comprehensive search strategy will be developed in collaboration with an experienced research librarian to maximize sensitivity and minimize the risk of missing relevant studies. However, this review has several limitations. While scoping reviews are designed to map the breadth of existing evidence rather than critically appraise study quality, the heterogeneity of study designs and variability in reporting completeness may limit the ability to draw firm conclusions regarding the relative importance of specific barriers and facilitators. Additionally, certain influential factors, such as implicit biases in clinical decision-making or subtle systemic barriers, may not be explicitly addressed or well-documented in the literature. As a result, critical but underreported determinants of OSA screening implementation may be overlooked. The absence of stakeholder consultation further limits the ability to capture context-specific or practical implementation challenges that are not reflected in published studies. These limitations should be taken into account when interpreting the findings.
