# Supplementary material for: Barriers to and facilitators of implementing obstructive sleep apnea screening in stroke patients: a scoping review protocol
Source: Front Neurol. 2025 Oct 16;16:1690372. doi: 10.3389/fneur.2025.1690372 (PMC12571605; doi:10.3389/fneur.2025.1690372)
Supplement: Supplementary file 1 [file Data_Sheet_1.ZIP › Additional file/Additional file 2.docx]

Supplementary Material

**Additional File 2.** Review search strategy.

Pubmed：

| **#** | **Search** |
| --- | --- |
| 1 | "Sleep Apnea, Obstructive"[MeSH Terms] |
| 2 | （Apneas，Obstructive Sleep［Title/Abstract］）OR（Obstructive Sleep Apneas［Title/Abstract］）OR（Sleep Apneas，Obstructive［Title/Abstract］）OR（Obstructive Sleep Apnea Syndrome［Title/Abstract］）OR（Obstructive Sleep Apnea［Title/Abstract］）OR（OSAHS［Title/Abstract］）OR（Syndrome，Sleep Apnea，Obstructive［Title/Abstract］）OR（Sleep Apnea Syndrome，Obstructive［Title/Abstract］）OR（Apnea，Obstructive Sleep［Title/Abstract］）OR（Sleep Apnea Hypopnea Syndrome［Title/Abstract］）OR（Syndrome，Obstructive Sleep Apnea［Title/Abstract］）OR（Upper Airway Resistance Sleep Apnea Syndrome［Title/Abstract］）OR（Syndrome，Upper Airway Resistance，Sleep Apnea［Title/Abstract］) OR (Sleep-Disordered Breathing [Title/Abstract]) OR (Sleep apnea syndrome [Title/Abstract]) |
| 3 | #1 OR #2 |
| 4 | "stroke"[MeSH Terms] OR "cerebral infarction"[MeSH Terms] OR "cerebral hemorrhage"[MeSH Terms] OR "cerebrovascular disorders"[MeSH Terms] |
| 5 | (stroke[Title/Abstract]) OR (cerebral infarction[Title/Abstract]) OR (cerebral hemorrhage[Title/Abstract]) OR (cerebrovascular events[Title/Abstract]) OR (cerebrovascular disease[Title/Abstract]) OR (cerebrovascular accident[Title/Abstract]) OR (apoplexy[Title/Abstract]) |
| 6 | #4 OR #5 |
| 7 | "mass screening"[MeSH Terms] OR "early diagnosis"[MeSH Terms] OR "risk assessment"[MeSH Terms] OR "polysomnography"[MeSH Terms] OR "surveys and questionnaires"[MeSH Terms] |
| 8 | "Screening"[Title/Abstract] OR "Diagnosis"[Title/Abstract] OR "Detection"[Title/Abstract] OR "Identification"[Title/Abstract] OR "Assessment"[Title/Abstract] OR "risk assessment"[Title/Abstract] OR "case finding"[Title/Abstract] OR "predictive model*"[Title/Abstract] OR "diagnostic tool*"[Title/Abstract] OR "sleep monitoring"[Title/Abstract] OR "STOP-BANG"[Title/Abstract] OR "berlin questionnaire"[Title/Abstract] OR "polysomnography"[Title/Abstract] OR "PSG"[Title/Abstract] OR "HSAT"[Title/Abstract] OR "home sleep apnea test"[Title/Abstract] |
| 9 | #7 OR #8 |
| 10 | "treatment outcome"[MeSH Terms] OR "attitude of health personnel"[MeSH Terms] OR "clinical decision making"[MeSH Terms] OR "treatment adherence and compliance"[MeSH Terms] OR "health knowledge, attitudes, practice"[MeSH Terms] |
| 11 | "health personnel attitude"[Title/Abstract] OR "healthcare provider knowledge"[Title/Abstract] OR "medical education"[Title/Abstract] OR "clinical decision making"[Title/Abstract] OR "training program*"[Title/Abstract] OR "health belief*"[Title/Abstract] OR "healthcare access"[Title/Abstract] OR "treatment outcome" [Title/Abstract] |
| 12 | #10 OR #11 |
| 13 | #9 OR #12 |
| 14 | #3 AND #6 AND #13 |
| No language, publication-type or date limits were applied to the searches. | |

CINAHL：

| **#** | **Search** |
| --- | --- |
| 1 | (MH "Sleep Apnea, Obstructive") OR (MH "Sleep Apnea Syndromes+") |
| 2 | TI ( "sleep apnea, obstructive" OR "Apneas，Obstructive Sleep" OR "Obstructive Sleep Apnea*" OR "Sleep Apneas，Obstructive" OR "Obstructive Sleep Apnea Syndrome" OR "OSAHS" OR "Sleep Apnea Hypopnea Syndrome" OR "Upper Airway Resistance Sleep Apnea Syndrome" OR "Sleep-Disordered Breathing" OR "Sleep apnea syndrome" ) OR AB ( "sleep apnea, obstructive" OR "Apneas，Obstructive Sleep" OR "Obstructive Sleep Apnea*" OR "Sleep Apneas，Obstructive" OR "Obstructive Sleep Apnea Syndrome" OR "OSAHS" OR "Sleep Apnea Hypopnea Syndrome" OR "Upper Airway Resistance Sleep Apnea Syndrome" OR "Sleep-Disordered Breathing" OR "Sleep apnea syndrome" ) |
| 3 | #1 OR #2 |
| 4 | (MH "Stroke+" OR MH "Cerebral Infarction" OR MH "Cerebral Hemorrhage+" OR MH "Cerebrovascular Disorders+" ) |
| 5 | TI ( "stroke" OR "cerebral infarction" OR "cerebral hemorrhage" OR "cerebrovascular disorder*" OR "cerebrovascular event*" OR "cerebrovascular disease" OR "cerebrovascular accident" OR "apoplexy" ) OR AB ( "stroke" OR "cerebral infarction" OR "cerebral hemorrhage" OR "cerebrovascular disorder*" OR "cerebrovascular event*" OR "cerebrovascular disease" OR "cerebrovascular accident" OR "apoplexy" ) |
| 6 | #4 OR #5 |
| 7 | (MH "Health Screening+") OR (MH "Diagnosis+") OR (MH "Nursing Assessment") OR (MH "Nursing Diagnosis") OR (MH "Polysomnography") OR MH "Surveys+") OR (MH "Questionnaires+") |
| 8 | TI ( "Screening" OR "Diagnosis" OR "Detection" OR "Identification" OR "Assessment" OR "Risk assessment" OR "Case finding" OR "Predictive model*" OR "Diagnostic tool*" OR "Sleep Monitoring" OR "STOP-BANG" OR "Berlin Questionnaire" OR "polysomnography" OR "HSAT" OR "Home Sleep Apnea Test" ) OR AB ( "Screening" OR "Diagnosis" OR "Detection" OR "Identification" OR "Assessment" OR "Risk assessment" OR "Case finding" OR "Predictive model*" OR "Diagnostic tool*" OR "Sleep Monitoring" OR "STOP-BANG" OR "Berlin Questionnaire" OR "polysomnography" OR "HSAT" OR "Home Sleep Apnea Test" ) |
| 9 | #7 OR #8 |
| 10 | (MH "Attitude of Health Personnel+") OR (MH "Decision Making, Clinical+") OR (MH "Guideline Adherence") OR (MH "Patient Compliance+") OR (MH "Medication Compliance") OR (MH "Treatment Outcomes+") OR (MH "Patient-Reported Outcomes+") |
| 11 | TI ( “Health Personnel Attitude“OR “Healthcare provider knowledge” OR “Medical education” OR “Training program*” OR “Clinical decision making” OR “Health belief*” OR “Healthcare access” OR “Patient Attitude*“ OR “Treatment outcome“ OR "Treatment Adherence and Compliance" ) OR AB ( “Health Personnel Attitude“OR “Healthcare provider knowledge” OR “Medical education” OR “Training program*” OR “Clinical decision making” OR “Health belief*” OR “Healthcare access” OR “Patient Attitude*“ OR “Treatment outcome“ OR "Treatment Adherence and Compliance" ) |
| 12 | #10 OR #11 |
| 13 | #9 OR #12 |
| 14 | #3 AND #6 AND #13 |
| No language, publication-type or date limits were applied to the searches. | |

CNKI:

SU=('卒中'+'脑梗死'+'脑出血'+'脑血管事件'+'脑血管疾病'+'脑血管意外'+'TIA') AND SU=('阻塞性睡眠呼吸暂停'+'阻塞性睡眠呼吸综合征'+'睡眠呼吸障碍'+'睡眠呼吸暂停'+'OSA'+'OSAS'+'OSAHS'+'打鼾') AND SU=('筛查'+'诊断'+'评估'+' STOP-BANG问卷'+'柏林问卷'+'多导睡眠监测'+'便携式监测'+'夜间血氧监测'+'知识'+'态度'+'教育'+'培训'+'课程'+'治疗结局'+'依从性'+'政策'+'资源可及性')

Grey literature:

The following search terms will be used in Google search engine. The first 200 results per query will be reviewed for each search.

| **#** | **Search** |
| --- | --- |
| 1 | ("obstructive sleep apnea" OR OSA) AND "stroke" AND screen* AND ("barrier*" OR "facilitat*"） |
| 2 | allintitle: "obstructive sleep apnea" stroke screening |
| 3 | ("obstructive sleep apnea" OR OSA) AND "stroke" AND (screen* OR detect* OR assess* OR evaluat*) |
| 4 | ("obstructive sleep apnea" OR OSA) AND "stroke" AND implement* |
| Exclusion of:   - Duplicate records — exact duplicates of records already captured from other sources - Commercial or promotional material - Job advertisements, course or training adverts | |

|  |
| --- |
