# Supplementary material for: Barriers to and facilitators of implementing obstructive sleep apnea screening in stroke patients: a scoping review protocol
Source: Front Neurol. 2025 Oct 16;16:1690372. doi: 10.3389/fneur.2025.1690372 (PMC12571605; doi:10.3389/fneur.2025.1690372)
Supplement: Supplementary file 1 [file Data_Sheet_1.ZIP › Additional file/Additional file 3.docx]

Supplementary Material

Records identified through database searching（n=）：CNKI（n=） WanFang（n=） Sinomed（n=） Web of Science（n=） PubMed（n=） CINAHL（n=） Embase（n=） Cochrane Library（n=）.

Additional records identified through other sources（n=）

Records included (n = )

Duplicate records removed (n = )

Records included (n = )

Read the title, abstract to exclude studies（n= ）

Records included (n = )

Read the full article to exclude（n= ）

Study design inconsistencies (n = )

Factors affecting OSA screening not addressed (n = )

etc.

Final inclusion of studies (n=)

**Identification of studies via databases and registers**

**Identification**

**Screening**

**Included**

**Additional File 3.** PRISMA flow diagram.
